# Sexual dysfunction among women of reproductive age: A systematic review and meta-analysis

**DOI:** 10.18502/ijrm.v19i5.9251

**Published:** 2021-06-23

**Authors:** Farzane Alidost, Reza Pakzad, Mahrokh Dolatian, Fatemeh Abdi

**Affiliations:** ^1^Department of Midwifery and Reproductive Health, School of Nursing and Midwifery, Tehran University of Medical Sciences, Tehran, Iran.; ^2^Department of Epidemiology, Faculty of Heath, Ilam University of Medical Sciences, Ilam, Iran.; ^3^Department of Midwifery and Reproductive Health, School of Nursing and Midwifery, Shahid Beheshti University of Medical Sciences, Tehran, Iran.; ^4^Department of Midwifery and Reproductive Health, School of Nursing and Midwifery, Alborz University of Medical Sciences, Karaj, Iran.

**Keywords:** Sexual dysfunction, Women, Reproductive age.

## Abstract

**Background:**

Available statistics show a high prevalence of sexual dysfunction (SD) among women worldwide. Various factors affect SD among women of reproductive age.

**Objective:**

To evaluate studies on the prevalence and determinants of SD in different parts of the world.

**Materials and Methods:**

MEDLINE, EMBASE, Web of Science, Scopus and ProQuest databases were systematically reviewed during 2000–2019. All original articles were reviewed. The STROBE checklist was used to evaluate the quality of the papers. I${}^{2}$ was calculated to determine heterogeneity. Fixed effects and/or random-effects models were applied to estimate the pooled prevalence. Meta-regression analysis was also performed to identify the sources of heterogeneity.

**Results:**

Based on the results of the meta-analysis (21 eligible studies), the pooled prevalence with 95% confidence interval of SD was estimated at 50.75% (41.73–59.78). The prevalence of pain and disorders in arousal, sexual desire, lubrication, orgasm, and sexual satisfaction were calculated (39.08%, 48.21%, 50.70%, 37.60%, 40.16%, and 35.02%, respectively). Also, age, depression, low education level, increased duration of the marriage, and the presence of chronic diseases were the highest risk factors for SD.

**Conclusion:**

The prevalence of SD in women of reproductive age varies in different countries. Considering the importance of female SD, further studies are needed to facilitate the development of relevant educational interventions.

## 1. Introduction

Sexual dysfunction (SD) is any dissatisfaction with one's sexual function which leads to distress. The diagnostic and statistical manual of mental disorders classifies female sexual dysfunction (FSD) as orgasmic, interest/arousal, and genito–pelvic pain/penetration disorders (1). Healthy sexual functioning is a major indicator of a healthy mental function. Negligence of sexual desire leaves irreparable effects on humans. The physical and psychological pressures caused by poor sexual satisfaction lead to sexual deviation and health problems (2). While sexuality is critically important in couples' marital satisfaction, sexual problems are inevitable in any marriage. They may negatively affect marital satisfaction, cause conflicts, and ultimately lead to divorce (3, 4). FSD is a common problem experienced by nearly 40–45% of women (5). According to a study conducted in Australia, 36% of women report at least one new SD in a span of 12 months (6). A similar rate (31.5%) was reported in a study of Iranian women aged 20–60 yr (7). In another study, it was reported as 20–40% (8). The existence of disorders in sexual desire, arousal, lubrication and orgasm in reproductive aged women has been documented by a previous study (9). Based on the available research, 30–60% of women experience SD at least once in their lives (4). A study on 821 women in Iran found the absence of sexual pleasure and orgasm in the sexual lives of 39% and 10.5% participants, respectively (10). Another study estimated the prevalence of anorgasmia in Iranian women at 27% (11). A variety of factors including mental health, sexual relationships, partner's sexual function, personality-related factors, duration of the relationship, infertility, drugs, chronic diseases, pelvic surgery, cancers, and postpartum changes can affect women's sexual function (12, 13). Other factors, such as hormonal changes, menstruation, lactation, menopause, and multiple births, may also have significant effects on women's sexual function (5). Also, the literature confirms that SD is associated with mental health problems, including depression and anxiety. This may be due to their lower ability to find an intimate partner, less social integration, and generally lower performance (14). Despite efforts to control sexual problems during the past decades, the existing statistics indicate the relatively unchanged high prevalence and extent of SD among women throughout the world (15). Considering the importance of sexual disorders as a health concern and an important factor affecting the quality of life of couples and the high and variable incidence of SD among reproductive-age women, this study used a meta-analysis to evaluate the prevalence and determinants of SD in Iran and other countries.

The aim of this study was to evaluate the prevalence of SD and its most important risk factors in women of reproductive age using the worldwide review studies.

## 2. Materials and Methods 

### Search strategy

The current study was a systematic review and meta-analysis reported based on the preferred reporting items for systematic reviews and meta-analyses (PRISMA) guidelines (16). The valid databases, such as, MEDLINE, Web of Science, EMBASE, Scopus, ProQuest, PubMed, and Google scholar were searched for combination keywords of “sexual dysfunction” OR “sexual disorder” OR “sexual problem” AND “women” OR “reproductive age women” OR “fertility age women.”

### Inclusion and exclusion criteria

The inclusion criteria were: (i) published studies in English or Persian between March 2000 and September 2019; (ii) using a cross-sectional, cohort, observational design to evaluate SD or its prevalence rate in women of the reproductive age (15–49 yr); and (iii) administrating the female sexual function index (FSFI) (total scores and scores of all domains) to measure SD. On the other hand, case reports, studies with incomplete data, studies using other questionnaires, studies performed on menopausal women, women with known psychiatric disorders, and individuals with chronic diseases were excluded.

### Study selection

All extracted articles were entered in Endnote X6 (Clarivate Analytics, Australia), and screening was done after removing duplicates. The screening was done in three steps. The titles and abstracts of all studies reviewed during the electronic and manual follow-up search process were assessed based on the inclusion criteria. The full-texts of relevant papers were examined based on the mentioned criteria. Blinding and task separation was applied in the study selection procedure. The inter-rater agreement was 87%.

### Quality assessment

The studies included in this review were assessed by two quality assessment methods given they had different study designs by RP and FA. The quality of studies was determined by evaluating their adherence to strengthening the reporting of observational studies in epidemiology (STROBE) checklist. Studies fulfilling all seven items, six items, and two or more items of the STROBE were classified to have high, medium, and low quality, respectively (17).

### Data extraction

Study selection was independently performed by two authors (FA, FA). The author's name, publication year, place, sample size, age, quality assessment score, prevalence of SD, and risk factors were extracted.

### Statistical analysis

Data were analyzed using the STATA software 14.0 (college station, Texas). The number of cases, prevalence of SD, and its different domains were derived. Heterogeneity was discovered using the Cochran's Q test of heterogeneity, and the I${}^{2}$ index was applied to quantify heterogeneity. I${}^{2}$ values > 0.7 were considered as high heterogeneity. The pooled prevalence with 95% confidence interval (CI) was calculated applying the “metaprop” command, and to calculate the pooled prevalence, the random-effects model was used (18). In addition, the meta-regression analysis was used to examine the effect of age and sample size as factors affecting heterogeneity among studies. The “metabias” command was applied to check the publication bias, and if there was any publication bias, the prevalence rate was adapted with the “metatrim” command using trim-and-fill method (19). In all analyses, a significance level of 0.05 was rated.

## 3. Results

**Figure 1:**
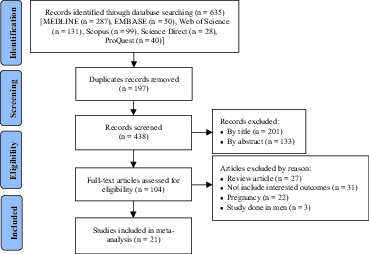
Search Flowchart.

Figure 1 shows the process of literature search. Overall, 635 studies were found through different databases. After excluding the redundant articles, 438 studies were retained. In the first stage of screening, 201 studies were rejected after reviewing the titles, which left us with 237 articles. Next, after reading abstracts, 133 studies were removed from the list. Then, the full-texts of the remaining 104 studies were reviewed, and 83 studies were excluded. Finally, a total of 21 studies (20–40) met the inclusion criteria and were deemed high quality in line with the STROBE checklist. Table I presents the characteristics of the included studies. The studies had different sample sizes (between 149 and 4,697) and considered 12,504 women in total. The study participants were from different geographic areas including Asia (n = 17), Africa (n = 3), and South America (n = 1). The prevalence of SD was reported in all studies. Table II shows the most important risk factors of SD in women of reproductive age (based on their frequency in the selected studies). These factors included age (n = 14), depression (n = 5), chronic diseases (n = 5), increased duration of marriage (n = 7), and low level of education (n = 10).

**Figure 2:**
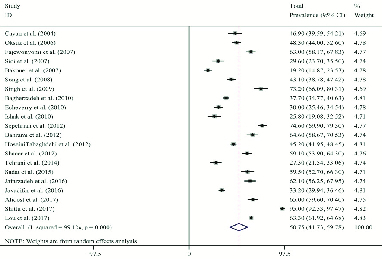
Forest plot of total sexual dysfunction prevalence. Each line segment indicates a 95% confidence interval. Diamond mark illustrates the pooled estimate and 95% confidence interval.

**Figure 3:**
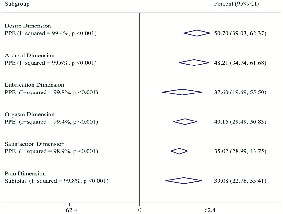
Pooled prevalence estimate of domains sexual dysfunction based on the random effects model. The diamond mark illustrates the pooled estimate for each domain.

**Figure 4:**
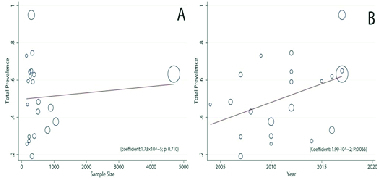
The relationship of the prevalence of sexual dysfunction with sample size (A) and the study publication year (B) based on meta-regression. The size of the circles shows the sample size. The prevalence rate of sexual dysfunction had no significant relationship with the study year or sample size. Appendix: Forest plot for the prevalence of different domains in sexual dysfunction including Desire, Arousal, Lubrication, Orgasm, Satisfaction, and Pain. Each line segment indicates a 95% confidence interval. Diamond mark illustrates the pooled estimate and 95% confidence interval.

The pooled results indicated the prevalence rate of SD as 50.75% (95% CI: 41.73–59.78) (Figure 2). The prevalence rates of desire, arousal, lubrication, orgasm, satisfaction, and pain were 50.7% (95% CI: 39.03–62.37), 48.21% (95% CI: 34.74–61.68), 37.60% (95% CI: 19.69–55.50), 40.16% (95% CI: 29.49–50.83), 35.02% (95% CI: 28.99–43.75), and 39.08% (95% CI: 22.76–55.41), respectively (Figure 3). Forest plot for all domains is provided in the appendix. According to the meta-regression analysis, relationships were found between the sample size and prevalence of SD in relational studies (coefficient: 1.73 × 10${}^{-5}$; 95% CI: –7.86 to 11.32 × 10${}^{-5}$; p = 0.710; Figure 4A). Meanwhile, evaluating the relationship between the publication year and the prevalence rate of SD showed an increasing trend in the prevalence over time. However, this increase was not statistically significant (coefficient: 1.99 × 10${}^{-2}$; 95% CI: –1.46 to 41.28 × 10${}^{-3}$; p = 0.066; Figure 4B). Based on our results, there is no publication bias for the total prevalence of SD and their domain.

**Table 1:** Summarized results of the included studies


**Author, year (Ref)**	**Place**	**QAS**	**SS**	**Total prevalence of SD,% (95% CI)**	**Number (%)**					
			**Desire**	**Arousal**	**Lubrication**	**Orgasm**	**Satisfaction**	**Pain**
**Alidost ** ***et al.*** **, 2017 (20)**	Asia	19	300	65% (60-70)	138 (46.3%)	159 (53%)	120 (40.7%)	144 (48.3%)	69 (22.7%)	144 (48.3%)
**Shittu ** ***et al.*** **, 2017 (21)**	Africa	15	300	95% (93-97)	273 (91.0%)	267 (89%)	297 (99.0%)	276 (92.0%)	258 (86.0%)	297 (99.0%)
**Fajewonyomi ** ***et al.*** **, 2007 (22)**	Africa	15	384	63% (58-68)	201 (83.0%)	130 (54%)	NR	154 (63.6%)	NR	55 (22.7%)
**Tehrani ** ***et al.*** **, 2014 (23)**	Asia	17	230	27% (22-33)	282 (35.6%)	313 (40%)	148 (18.9%)	211 (27.3%)	118 (15.2%)	439 (56.1%)
**Sidi ** ***et al.*** **, 2007 (24)**	Asia	17	230	30% (24-35)	NR	140 (61%)	116 (50.4%)	136 (59.1%)	120 (52.2%)	156 (67.8%)
**Oksuz ** ***et al.*** **, 2006 (25)**	Asia	14	518	48% (44-53)	250(48.3%)	186 (36.9%)	212 (40.9%)	221 (42.7%)	233 (45.0%)	222 (42.9%)
**Çayan ** ***et al.*** **, 2004 (26)**	Asia	15	179	47% (40-54)	108 (60.3%)	77 (43.0%)	68 (38.0%)	82 (45.8%)	68 (38.0%)	65 (36.8%)
**Jafarzadeh ** ***et al.*** **, 2016 (27)**	Asia	16	264	62% (56-68)	129 (49.2%)	114 (43.2%)	95 (36.0%)	101 (38.6%)	69 (26.1%)	93 (35.2%)
**Javadifar ** ***et al.*** **, 2016 (28)**	Asia	15	800	33% (30-36)	NR	98 (24.5%)	90 (22.5%)	72 (18.0%)	96 (24.2%)	115 (28.8%)
**Sepehrian ** ***et al.*** **, 2012 (29)**	Asia	17	330	75% (70-79)	NR	142 (42.3%)	NR	NR	76 (23.0%)	158 (47.7%)
**Sadat ** ***et al.*** **, 2015 (30)**	Asia	17	200	60% (53-66)	78 (39.0%)	74 (37.0%)	57 (28.8%)	49 (24.5%)	45 (22.5%)	39 (19.5%)
**Bagherzadeh ** ***et al.*** **, 2010 (31)**	Asia	18	1054	38% (35-41)	749 (71.1%)	700 (66.4%)	400 (38.0%)	366 (34.8%)	264 (24.9%)	405 (38.4%)
**Lou ** ***et al.*** **, 2017 (32)**	Asia	17	4697	63% (62-65)	2361 (46.5%)	4019 (80.1%)	1628 (32.4%)	1507 (29.0%)	1522 (30.3%)	1587 (31.6%)
**Bahrami ** ***et al.*** **, 2012 (33)**	Asia	11	250	65% (59-71)	10 (4.01%)	10 (4.22%)	13 (4.61%)	10 (4.43%)	NR	13 (5.2%)
**Hoseini Tabaghdehi ** ***et al.*** **, 2012 (34)**	Asia	12	899	45% (42-48)	360 (39.6%)	315 (35.5%)	360 (39.8%)	386 (42.7%)	NR	422 (47.3%)
**Bakouei ** ***et al.*** **, 2007 (35)**	Asia	14	318	19% (15-24)	152 (48.4%)	127 (40.3%)	38 (12.0%)	60 (18.6%)	35 (11.3%)	64 (19.8%)
**Shaeer ** ***et al.*** **, 2012 (36)**	Africa	16	344	59% (54-64)	134 (39.0%)	NR	72 (20.7%)	NR	NR	65 (19.1%)
**Echeverry ** ***et al.*** **, 2010 (37)**	SA	15	391	30% (25-35)	NR	NR	NR	NR	NR	NR
**Singh ** ***et al.*** **, 2009 (38)**	Asia	16	149	73% (66-80)	115 (77.2%)	136 (91.3%)	144 (96.6%)	129 (86.0%)	121 (81.2%)	96 (64.4%)
**Song ** ***et al.*** **, 2008 (39)**	Asia	18	504	4% (39-47)	221 (44.0%)	247 (49.0%)	186 (37.0%)	161 (32.0%)	186 (37.0%)	176 (34.6%)
**Ishak ** ***et al.*** **, 2010 (40)**	Asia	16	163	26% (19-33)	64 (39.3%)	42 (25.8%)	35 (21.5%)	27 (16%)	35 (21.5%)	27 (16.6%)
SD: Sexual dysfunction, SA: South America, NR: Not reported, QAS: Quality assessment score, SS: Sample size, CI: Confidence interval										

**Table 2:** Risk factors of SD in women of reproductive age


**Author, year (Ref)**	**Country**	**Age (Range/average)**	**Results (Risk factors)**
**Alidost ** ***et al.*** **, 2017 (20)**	Iran	27.38 ± 5.49	-Quality of life -Age (directly) -Prenatal anxiety -Income (indirectly through the quality of life)
**Shittu ** ***et al.,*** ** 2017 (21)**	Nigeria	15-49	-Aging
**Fajewonyomi ** ***et al.,*** ** 2007 (22)**	Nigeria	21-45	-Various illnesses (medical, surgical, psychiatric, and gynecological problems) -History of sexual abuse -The polygamous family type -Emotionally unstable women
**Tehrani ** ***et al.,*** ** 2014 (23)**	Iran	33.5 ± 6.94	-Length of married life -Perceived interest of the spouse -Total satisfaction with pattern life -Ability to express sexual desires in women's
**Sidi ** ***et al.,*** ** 2007 (24)**	Malaysia	39.2 ± 10.5	-Age (< 45 yr old) -Predominantly Malay -Having intercourse < 3 times a week -Married for 14 yr or longer -Having at least four children -Married to an older spouse (aged > 42 yr) -Premenopausal stage -Higher academic status in women
**Oksuz ** ***et al.,*** ** 2006 (25)**	Turkey	30 ± 8.5	-Age -Smoking -Menopause -Diet -Marital status
**Çayan ** ***et al.,*** ** 2004 (26)**	Turkey	40.3 ± 11.7	-Older age -Lower level of education -Unemployment -Chronic disease -Multiparity -Menopause
**Jafarzadeh ** ***et al.,*** ** 2016 (27)**	Iran	32.2 ± 10.27	-Age -Level of education (person and spouse) -Number of children -Length of married life -Consanguinity -Medications
**Javadifar ** ***et al.,*** ** 2016 (28)**	Iran	29.57 ± 7.59 in rural and 30.73 ± 7.11 in urban areas	-Type of delivery (normal vaginal delivery) -No delivery (the base delivery) -Normal physical profile -Place of residence
**Sepehrian ** ***et al.,*** ** 2012 (29)**	Iran	28.44 ± 7.58	-Depression -Anxiety -Stress
**Sadat ** ***et al.,*** ** 2015 (30)**	Iran	30.52 ± 7.95 (women with FSD) 28.47 ± 6.16 (women without FSD)	-Depression -Anxiety -Stress -Low level of education -Older age -Longer duration of the marriage
**Bagherzadeh ** ***et al.,*** ** 2010 (31)**	Iran	35.78 ± 11.88	-Age of subjects -Husbands' age
**Author, year (Ref)**	**Country**	**Age (Range/average)**	**Results (Risk factors)**
**Lou ** ***et al.,*** ** 2017 (32)**	China	20-60	-Age -Spouse's sexual ability -Poor marital kindness -Spouse's sexual dysfunction -Dissatisfaction with married life -Living in a rural area -Chronic pelvic pain -Chronic disease -Previous pelvic surgery -Vaginal delivery -Lower education -Postmenopausal
**Bahrami ** ***et al.,*** ** 2012 (33)**	Iran	34.7 ± 6.4	-Sexual satisfaction -Age -Marriage age -Educational level
**Hoseini Tabaghdehi ** ***et al.,*** ** 2012 (34)**	Iran	28.37 ± 6.06	-Age of menarche -Fear of pregnancy
**Bakouei ** ***et al.,*** ** 2007 (35)**	Iran	28	-Education level -Partner's job -Economic status -Number of children -Contraception method -Chronic disease -Frequency of intercourse
**Shaeer ** ***et al.,*** ** 2012 (36)**	Egypt	28.9 ± 5.9	-Depression -Insufficient foreplay -Practice of masturbation -Erectile dysfunction in the male partner -Premature ejaculation in the male partner -Dissatisfaction with partner's penile size -Cycle duration -Education level
**Echeverry ** ***et al.,*** ** 2010 (37)**	Colombia	18-40	-Lower education -Depressive feelings -Antidepressants
**Singh ** ***et al.,*** ** 2009 (38)**	India	38.2 ± 10.7	-Age > 40 yr -Fewer yr of education -Monthly income -Years since marriage
**Song ** ***et al.,*** ** 2008 (39)**	Korea	28.5 ± 6.7	-Increasing age -Low frequency of sexual intercourse -Depression -History of sexual abuse -Homosexuality
**Ishak ** ***et al.,*** ** 2010 (40)**	Malaysia	44 ± 10.4	-Age -Husband's age -Duration of married life -Medical illness -Menopause -Frequency of sexual intercourse

## 4. Discussion

Sexual relations are essential for human survival and reproduction and have major spiritual and cultural connotations. According to the World Health Organization, SD is a “disorder in sexual desire and the psychophysiological transforms that defined the sexual response cycle and which results in signed distress and relational problem” (41). SD may involve dyspareunia, sexual desire and arousal disorders, and orgasmic dysfunction. These are all major public health issues with considerable negative effects on a person's daily life (42). Nationwide policies to resolve such issues cannot be developed unless the prevalence rate of SD is known. An increasing number of population studies have evaluated FSD under various cultural settings during the past decade. The aim of this systematic review is to provide an overview of the prevalence of FSD among women of reproductive age in different countries, cultural backgrounds, and age groups. It is hoped that the results would better clarify the effects of FSD on women's lives. Since the sexual behavior in Iran has become a taboo due to the historical, cultural, and religious reason and religious prohibitions, it isn't easy for women to talk about it; so dealing with the issue of sexual needs has always been accompanied by shame and anxiety. Further studies in this field are necessary to better understand the challenges (9).

While a larger number of studies were available, they were not included in the analysis as they had applied other tools for the assessment of FSD or did not have an accessible full-text. Moreover, only studies on samples of women have entered the analysis. Based on the obtained results, the FSD had a very high prevalence rate and affected about 51% of women. A meta-analysis by Hosseini Tabaghdehi and coworkers reported the prevalence rate of FSD as 48% (34). In a review study, Aggarwal and coworkers calculated the prevalence rate of SD as 55.6% (43).

In this study, the arousal disorder had the greatest prevalence (about 48%). Pain and disorders in sexual desire, lubrication, orgasm, and sexual satisfaction had prevalence rates of 39%, 51%, 38%, 40%, and 35%, respectively. Ramezani and coworkers found disorder in sexual desire as the most prevalent (65.8%) form of SD. They reported the prevalence rates of sexual pain, arousal disorder, and orgasmic disorder as 35.2%, 59.6%, and 35.2%, respectively (9). In a review study, Aggarwal and coworkers highlighted the orgasmic disorder as the most prevalent (91.7%) form of SD. Moreover, lubrication disorder affected 19% of the women (43).

In the present study, increasing age and duration of marriage increased the prevalence of different forms of FSD, that is, pain and disorders in desire, arousal, lubrication, orgasm, and satisfaction. Also, the increase in age affects the sexual response cycle and the physiology of marital affection and creates hormonal changes. As a result, sexual desire and frequency decrease, which ultimately leads to a reduction in marital satisfaction (44).

Other factors increasing the prevalence of FSD included depression, low education, and chronic illnesses. Convery and coworkers reported that women with a higher educational level had a lower SD and attributed this to reasons such as increased awareness and less negative attitudes (45). Also, chronic diseases due to decreased physical strength, reduced ability to perform daily activities, hospitalization, and, eventually, depression caused by the disease can be a major contributors to sexual problems (46). On the other hand, depressed people experience persistent insensibility, more frustration, helplessness, worthless, guilt, and generally lose their attachment to life, work, and even sex (47).

In this meta-analysis, we only focused on studies evaluating the prevalence of SD in women of reproductive age. While the different prevalence of FSD in various populations may demonstrate the effects of conditions (e.g., culture) on sexual problems, they may also be caused by women's unwillingness to discuss their sexual problems, their perception of sexual problems, and the prevailing sexual culture in different countries. Nevertheless, considering the high prevalence of SD in many countries including Iran, and since SD has a significant impact on marital satisfaction, the intimacy between couples and their quality of life, healthcare providers are recommended to provide women of reproductive age with SD-related advice and counseling to promote public health and marital satisfaction. A limitation of this study was collecting data from cross-sectional studies performed only on women of reproductive age. Moreover, as women might be unwilling to respond to questions about their private sexual life, the results obtained by the reviewed studies may be inaccurate. Furthermore, only studies using the FSFI were included in this systematic review and based on this questionnaire, we classified SD into six domains (hypoactive desire disorder, arousal disorder, orgasmic disorder, dyspareunia, lubrication disorder, and satisfaction disorder). The diagnostic and statistical manual of mental disorders (DSM5) has classified SD into three domains: sexual interest/arousal, genito–pelvic pain/penetration, and orgasmic disorder. This may be the most important limitation of our study. Finally, the education level of the respondents (which might have affected their responses to the items in the questionnaire) was not considered in the selected studies. One of the strengths of this study is an exclusive review of studies that only used the FSFI questionnaire, as well as the examination of studies that were conducted only on women of reproductive age, which reduces the bias.

## 5. Conclusion

The prevalence of SD varies in women of reproductive age in different countries. Considering the importance of female SD, further studies are needed to facilitate the development of relevant educational interventions.

##  Conflict of Interest

The authors declare that they have no conflict of interest.
